# A Retrospective Study on the Prevalence of Hepatitis A Virus in Patients With Acute Viral Hepatitis at a Tertiary Care Hospital

**DOI:** 10.7759/cureus.78676

**Published:** 2025-02-07

**Authors:** T Anitha, P Sivagamasundari, Eunice Swarna Jacob, P B Praveen Kumar

**Affiliations:** 1 Department of Microbiology, Thanjavur Medical College, Thanjavur, IND

**Keywords:** acute viral hepatitis, elisa, hav, public health policies, young children

## Abstract

Hepatitis A virus (HAV) is a common cause of acute viral hepatitis, especially in developing countries. HAV poses a major public health problem in India. A retrospective study was carried out to ascertain the prevalence, demographic characteristics, and clinical outcomes of the hepatitis A virus in patients who presented with acute viral hepatitis at a tertiary care hospital between November 2022 and October 2024. About 656 samples were included in this study; 142 of the 656 samples tested positive for HAV IgM enzyme-linked immunosorbent assay (ELISA). In July and August of 2023, there was a sharp increase in seropositive cases linked to person-to-person transmission. In this study, the overall prevalence was 22%. The most frequent symptoms were fever, followed by stomach pain and yellowish urine. The hepatitis A virus primarily affects young children and a small percentage of adults and adolescents from lower and intermediate socioeconomic backgrounds who drink subterranean water. The seroprevalence of the hepatitis A virus is influenced by time and geographic spread. When caring for children under two, the most important preventative measure is to wash your hands properly. The current study also emphasizes the necessity of planning decisions on public health policies, such as vaccination plans, safe drinking water sources, and sanitation programs, in order to better control the hepatitis A virus and ensure its appropriate usage.

## Introduction

Hepatitis A virus (HAV) is a 27 nm, non-enveloped, single-stranded RNA virus, a member of the Picornaviridae family and genus Hepatovirus [1]. Both sporadic and epidemic forms of acute hepatitis are caused by the hepatitis A virus, which is mostly spread by the fecal-oral route through the intake of contaminated food and drink [1]. While adults develop more severe liver diseases, including cholestatic and relapsing hepatitis, which have a longer course, infected children typically show no signs or very moderate constitutional symptoms [1].

In developing nations, acute viral hepatitis is a significant health issue, particularly in rural and slum communities with poor socioeconomic levels [2]. People are impacted by inadequate sanitation, personal hygiene, and a shortage of safe drinking water [2].

The WHO estimates that 1.4 million new cases of the hepatitis A virus are detected worldwide each year [2]. The age of the infected person mostly impacts the severity and clinical prognosis of the HAV infection [3]. The present study was conducted to determine the seroprevalence of hepatitis A virus infection, analyze the demographic patterns, and assess clinical presentations in patients with acute viral hepatitis at a tertiary care hospital.

The overall prevalence of the hepatitis A virus in this study was 22% (n=142). The proper diagnosis may only be made by analyzing patients’ sera for the presence of particular anti-viral antigens or antibodies [4]. The best control strategies must stop infected people from fecal contamination before their clinical illness manifests.

This article was previously presented as an oral presentation on April 11, 2023, at the Artificial Intelligence and Infectious Diseases Continuing Medical Education, 2023

## Materials and methods

Study design and place

This was a hospital-based, retrospective cross-sectional study carried out from November 2022 to October 2024 at Thanjavur Medical College Hospital, India.

Inclusion criteria

Patients of all age groups from various outpatient departments and wards, with clinical signs and symptoms like fever, vomiting, abdominal pain, yellowish discoloration of eyes and dark-colored urine, and loss of appetite, and laboratory findings suggestive of acute viral hepatitis, were included in this study.

Exclusion criteria

Patients with a history of preexisting liver disease were excluded from the study. Patients with confirmed cases of other types of viral hepatitis (hepatitis B, C, or E) and repeat samples from the same patient during the study period were likely excluded.

Sample collection

Patients who were presented with acute viral hepatitis and demographic characteristics, including age, gender, area of residence, socioeconomic status, and source of drinking water, were elicited and recorded in the case record form. Under strict aseptic precautions, 5 ml of venous blood was collected in a sterile vacutainer after obtaining informed consent from the patients for the assessment of anti-HAV IgM antibodies.

Sample rejection criteria

Following appropriate discussion with the clinical team, serum samples that were received in unsuitable containers and those that were either wrongly labeled or unlabeled were summarily rejected.

Sample processing

On a routine basis, blood samples were received and allowed to clot naturally. The serum was separated from the collected blood samples after centrifugation at 2000 revolutions per minute (rpm) for 20 minutes. It is then aliquoted into separate vials and labeled accordingly. A commercially available enzyme-linked immunosorbent assay (ELISA) test kit (Dia.Pro, Milan, Italy) was used to analyze anti-HAV IgM in accordance with the manufacturer's instructions. Each test run was conducted using the proper calibrators. A calibrated ELISA reader was used to determine the optical density (OD). As directed by the manufacturer, the test run's validity and the outcomes were examined. A negative result indicates that there are no detectable anti-HAV IgM antibodies in the sample and the patient is not undergoing an acute infection by the hepatitis A virus. Any patient showing an equivocal result should be retested by examining a second sample after one to two weeks from the first testing. A positive result is indicative of hepatitis A virus infection, and therefore the patient should be treated accordingly. The results were documented and analyzed statistically.

Data analysis

The results were documented and analyzed by using descriptive statistics, such as frequency counts and percentages.

The samples were obtained from the patients, including both outpatient departments and wards, who were presented with symptoms suggestive of acute viral hepatitis and processed at the microbiology laboratory. The requisition form received in the microbiology laboratory was used as a data collection tool to document patient details such as age, gender, area of residence, and diagnosis. We collected other patient-related data, like symptoms, socioeconomic status, and source of drinking water, from hospital medical records, the patient case sheet, and through telephone conversations. The information collected from each patient was reported and reorganized with the help of Microsoft Excel (Microsoft Corporation, Redmond, Washington, United States) spreadsheets.

Descriptive analysis was done by breaking down the patients into different categories, such as age groups and gender, and summarizing the number of positive cases in each group, and analysis was done through summary tables. The demographic characteristics were analyzed by calculating percentages. A seasonal variation in cases was analyzed through segmentation of month-wise data and comparison of case counts.

Ethical considerations

Since this is a retrospective study, broader consent, and assent were obtained from patients and patient caregivers, respectively, at the time of data collection through telephone conversations, indicating potential future research uses of their data.

## Results

A total of 656 blood samples were received for microbiological analysis. Out of 656 samples tested, 22% (n=142) were tested positive for anti-HAV IgM. Anti-HAV IgM seropositivity was 23% (n=55) in females whereas, it was 20.8% (n=87) in males. Age-wise distribution of subjects showed high seropositivity. Around 28.3% (n=34) in ≤10 years of age group, followed by 11-20 years (23.7%, n=72), 21-30 years (18.6%, n=30), and those more than 30 years (8.4%, n=6) (Table 1). There was a sudden rise in seropositive cases during July and August of 2023 associated with person-to-person transmission.

**Table 1:** Prevalence of hepatitis A virus (HAV) by age group and sex distribution

Age group in years	Males		Females		Total Positives/Tested	% Positive anti-hepatitis A virus
	Positives/Tested	% Positive anti-HAV	Positives/Tested	% Positive anti-HAV		
≤ 10	23/95	24.2	11/25	44.0	34/120	28.3
11-20	44/184	23.9	28/120	23.3	72/304	23.7
21-30	15/85	17.6	15/76	19.7	30/161	18.6
More than 30	5/53	9.4	1/18	5.5	6/71	8.4
Total	87/417	20.8	55/239	23.0	142/656	21.6

Infections with viruses are more prevalent in rural areas compared to urban areas. According to the modified Kuppuswamy classification, there were 16.5%, 27.9%, 33.1%, 16.9%, and 8.7% people affected, representing lower, upper-lower, lower-middle, upper-middle, and higher socioeconomic status, respectively. The most common source of drinking water in the household was underground boring water (25.5%, n=81), followed by municipal supply (21.2%, n=43) and bottled water (13.2%, n=18) (Table 2).

**Table 2:** Demographic characteristics

Characteristics	Positives/Tested	% of people with hepatitis A virus
Area of residence		
Rural	79/339	23.3
Urban	63/317	19.9
Socioeconomic status		
Lower	21/127	16.5
Upper-lower	36/129	27.9
Lower-middle	51/154	33.1
Upper-middle	26/154	16.9
Higher	8/92	8.7
Source of drinking water		
Underground water	81/317	25.5
Municipal supply	43/203	21.2
Bottled water	18/136	13.2

The common presenting complaints were fever (99%, n=650), followed by malaise (97.8%, n=642), abdominal pain (94%, n=617), yellowish discoloration of eyes (93.2%, n=612), dark-colored urine (93%, n=610), and loss of appetite (89%, n=584).

Hepatitis A cases were commonly observed in July and August of every year associated with person-to-person transmission (Table 3).

**Table 3:** Seasonal variation of seropositive cases of hepatitis A

Month and year	Total cases	Total positive cases
November 2022 to December 2023	281	82
January 2024 to October 2024	375	60
Total	656	142

## Discussion

Hepatitis A is often linked to recurrent jaundice and is one of the most common causes of infectious jaundice worldwide. As nations raise their standards of personal hygiene and sanitation, the geographic distribution of HAV infection seems to be shifting, according to the incidence rates from confirmed acute HAV cases. No matter how the infection is contracted, the hepatitis A virus takes 10 to 50 days to incubate, with a typical incubation period of about one month [5].

Despite being rather stable in a range of environmental circumstances, the hepatitis A virus can be rendered inactive by standard disinfectants such as bleach and hypochlorite. The main way that HAV is spread globally is from person to person through the fecal-oral pathway. Children were the main source of person-to-person transmission prior to the development of the HAV vaccine. Although hepatitis A is usually an acute, self-limiting illness, the way it manifests clinically changes with age [6].

Recurrent epidemics are a common occurrence. The hepatitis A virus typically causes sudden, explosive outbreaks when feces contaminate a single source, such as milk, food, or drinking water. Very seldom is HAV spread via administering blood or by using tainted syringes and needles [7].

The overall prevalence of the hepatitis A virus in the current study was found to be 22%. A study conducted in Mangalore reported 19.3% seropositivity for HAV in the patients presenting with acute viral hepatitis [1]. In central Karnataka, a study done by Manjunatha Sarthi et al. in 2016 reported a prevalence of 37.25% [1]. Seropositivity for HAV was higher in females compared to males in this study. Many investigators have reported the male preponderance for HAV infection in their studies. In this study, positivity for anti-HAV was found to be high (28.3%) in children under 15 years of age. This is in agreement with other earlier studies. A similar age group was more affected by acute hepatitis in this study by Manjunatha Sarthi et al. [1]. In this current study, the most common presenting complaint was fever. Similar studies were conducted by Javaria Rasheed et al. in Multan, Pakistan [2]. The prevalence of hepatitis A was found to be 14.55% by Meghna S. Palewar et al. in 2022 [8]. When fecal HAV shedding is still happening and serum aminotransferase activity is high during acute sickness, anti-HAV antibodies can be found. Rarely lasting six to twelve months, this early antibody response is primarily of the IgM class. However, IgG-class anti-HAV antibodies take over as the most common antibody after convalescence. Hepatitis A does not develop into chronic liver disease; it is still self-limiting. In this study, the seroprevalence of the hepatitis A virus was 4.29% [9]. Similarly, a study done by A. Muneer et al. reported that the seroprevalence of the hepatitis A virus was 60% [10]. A study done by Bing-Yu Yan et al. between 2006 and 2014 provided a comparison of seroprevalence before and after vaccination [11]. In the rural Amazon in 2004, a comparative study done with the hepatitis E virus showed seropositivity of 82.9% [12]. A similar study done by Pooja Semwall et al. revealed a seropositivity of 66.92% [13]. In Uttarakhand in 2020, a study was done on children, which revealed seropositivity of 85.2% [14]. In 2021, a study done by Geetika Rana et al. showed seropositivity at 48.93% [15]. Hepatitis A is rarely fatal, and studies have shown that fatality rates among cases of co-infection with hepatitis B were up to 5.6 times higher compared to patients without co-infection [16].

The best control strategies must stop infected people from fecal contamination before their clinical illness manifests. Therefore, when caring for children under two, the most important control measures are to wash your hands properly and refrain from using work practices that make it easier for your hands to get contaminated. The immunization is safe, efficacious, and advised for use in individuals older than one year. Excellent immunogenicity is seen in adults, adolescents, and children aged one year or older, with protective antibody levels (>= 20 m IU/mL) [5]. Large pools of normal adult plasma are used to make immune gamma globulin, which, when administered within one to two weeks following hepatitis A exposure, provides passive protection to around 90% of people exposed. Immunoglobulin should be replaced by HAV vaccinations, which provide longer-lasting immunity [7].

Limitations and strength

This study was conducted in one tertiary care hospital, so the findings may not be generalizable to other regions or healthcare settings. The study does not track long-term outcomes for seropositive patients, limiting insights into chronic impacts or recurrence rates. Other factors like co-infections and nutritional status were not analyzed in-depth. Additionally, because of the retrospective study design, it may limit the accuracy or completeness of records. With large samples analyzed, the study provides a robust dataset, enhancing the reliability of its findings. By reviewing data from a two-year period, the study captures trends over time, including seasonal variations like the July-August spike in cases. The findings emphasize actionable public health measures, such as vaccination programs, sanitation, and safe water practices, which could directly benefit public health policies.

## Conclusions

In the present study, HAV seroprevalence was found to be 22%. Hepatitis A virus causes acute viral hepatitis most commonly in early childhood, with a small number of adolescents and adults. Females are more commonly infected than males. The most critical control measure is proper handwashing. The present study also highlights the need for planning decisions on public health policies, including sanitation programs as well as the formulation of vaccination strategies for appropriate use and better control of hepatitis A.
